# Bioclipse 2: A scriptable integration platform for the life sciences

**DOI:** 10.1186/1471-2105-10-397

**Published:** 2009-12-03

**Authors:** Ola Spjuth, Jonathan Alvarsson, Arvid Berg, Martin Eklund, Stefan Kuhn, Carl Mäsak, Gilleain Torrance, Johannes Wagener, Egon L Willighagen, Christoph Steinbeck, Jarl ES Wikberg

**Affiliations:** 1 Department of Pharmaceutical Biosciences, Uppsala University, Uppsala, Sweden; 2 Wellcome Trust Genome Campus, European Bioinformatics Institute, Hinxton, UK; 3 Max von Pettenkofer-Institut, Ludwig-Maximilians-Universität, Munich, Germany

## Abstract

**Background:**

Contemporary biological research integrates neighboring scientific domains to answer complex questions in fields such as systems biology and drug discovery. This calls for tools that are intuitive to use, yet flexible to adapt to new tasks.

**Results:**

Bioclipse is a free, open source workbench with advanced features for the life sciences. Version 2.0 constitutes a complete rewrite of Bioclipse, and delivers a stable, scalable integration platform for developers and an intuitive workbench for end users. All functionality is available both from the graphical user interface and from a built-in novel domain-specific language, supporting the scientist in interdisciplinary research and reproducible analyses through advanced visualization of the inputs and the results. New components for Bioclipse 2 include a rewritten editor for chemical structures, a table for multiple molecules that supports gigabyte-sized files, as well as a graphical editor for sequences and alignments.

**Conclusion:**

Bioclipse 2 is equipped with advanced tools required to carry out complex analysis in the fields of bio- and cheminformatics. Developed as a Rich Client based on Eclipse, Bioclipse 2 leverages on today's powerful desktop computers for providing a responsive user interface, but also takes full advantage of the Web and networked (Web/Cloud) services for more demanding calculations or retrieval of data. The fact that Bioclipse 2 is based on an advanced and widely used service platform ensures wide extensibility, making it easy to add new algorithms, visualizations, as well as scripting commands. The intuitive tools for end users and the extensible architecture make Bioclipse 2 ideal for interdisciplinary and integrative research.

Bioclipse 2 is released under the Eclipse Public License (EPL), a flexible open source license that allows additional plugins to be of any license. Bioclipse 2 is implemented in Java and supported on all major platforms; Source code and binaries are freely available at http://www.bioclipse.net.

## Background

Contemporary biological research integrates neighboring scientific domains to answer complex questions in fields such as systems biology and drug discovery [1]. To this end, researchers combine diverse types of data from increasingly available public sources. This calls for flexible analysis tools which can quickly be adapted to new and unforseen tasks through open interfaces and extensible mechanisms. Workflows have been proposed as one solution for this problem, and have been shown to perform well in several studies. While orchestration tools like Taverna [2] are well equipped for producing workflows with reproducible and reusable functionality, a workbench is focused on iterative science where analysis and visualization tools are available for data exploration. In order to meet the demands of today's data-intensive problem settings, a workbench must be able to automate operations on large scale and produce executive summaries of analyses.

The Bioclipse workbench [3], presented here in an improved version, constitutes such a flexible analysis framework. It is a free, open source workbench that allows users to work with resources and entities in the life sciences, such as chemical structures, sequences, spectra, and alignments. Following its first release in early 2007, Bioclipse has, as of September 2009, been downloaded more than 30 000 times, and been awarded 3 international prizes for its innovative architecture. Bioclipse 2 constitutes a complete rewrite which provides the project with a strong foundation for integrating life science components, and turns Bioclipse into a stable, scalable platform for the life sciences. A major update from previous versions is that Bioclipse 2 is completely scriptable, allowing to scale up analyses by automating functionality in the Bioclipse Scripting Language (BSL) and also enables sharing of reproducible scripts.

## Implementation

Bioclipse is built in Java on the Eclipse Rich Client Platform (RCP) http://www.eclipse.org/rcp which has a component-based architecture where all components (including the core ones) are referred to as plugins. The plugin architecture builds on the OSGi framework [4], which is a dynamic Java-based component model. There is an entire ecosystem of Eclipse plugins available in various domains such as mathematics, finance, education, and software development. While taking advantage of the existing plugins, Bioclipse extends this ecosystem with a domain object model and implementations for the life sciences. It is very easy to contribute new plugins for Bioclipse, and tutorials are available on the Bioclipse wiki. While the integration with the Bioclipse platform needs to be written in Java, the plugins' functionality can be implemented in any language. The use of a standardized plugin architecture is a major advantage over many existing platforms in the life sciences who provide their own implementations. In fact, prominent applications such as Taverna [2] and Cytoscape [5] have recently announced that future versions will be based on OSGi due to its many advantages.

In Bioclipse 2, all functional code contributed by plugins is structured in *Bioclipse Managers*; e.g. code that provides access to 3D conformer generation using Balloon [6] are available in the BalloonManager. Bioclipse 2 makes use of Spring http://www.springsource.org to provide dependency injection and aspect oriented programming (AOP), which allows for encapsulating concerns into separate entities. Bioclipse 2 makes us of this to inspect Manager calls and decide e.g. if calls should be run in separate threads, and also records method usage. Annotated Manager objects are published into the scripting environment, and hence the same objects that are called from the GUI are reachable from scripts.

The life sciences make extensive use of the Web as a medium for distributing data and software tools. As a Rich Client, Bioclipse takes full advantage of this service-oriented architecture (SOA), and provides a platform to integrate Web services using various technologies, including SOAP, REST, and XMPP + IO Data [7]. The latter is a novel technology which enables truly asynchronous communication between the client and server.

## Results and Discussions

### Cheminformatics

**Figure 1:**
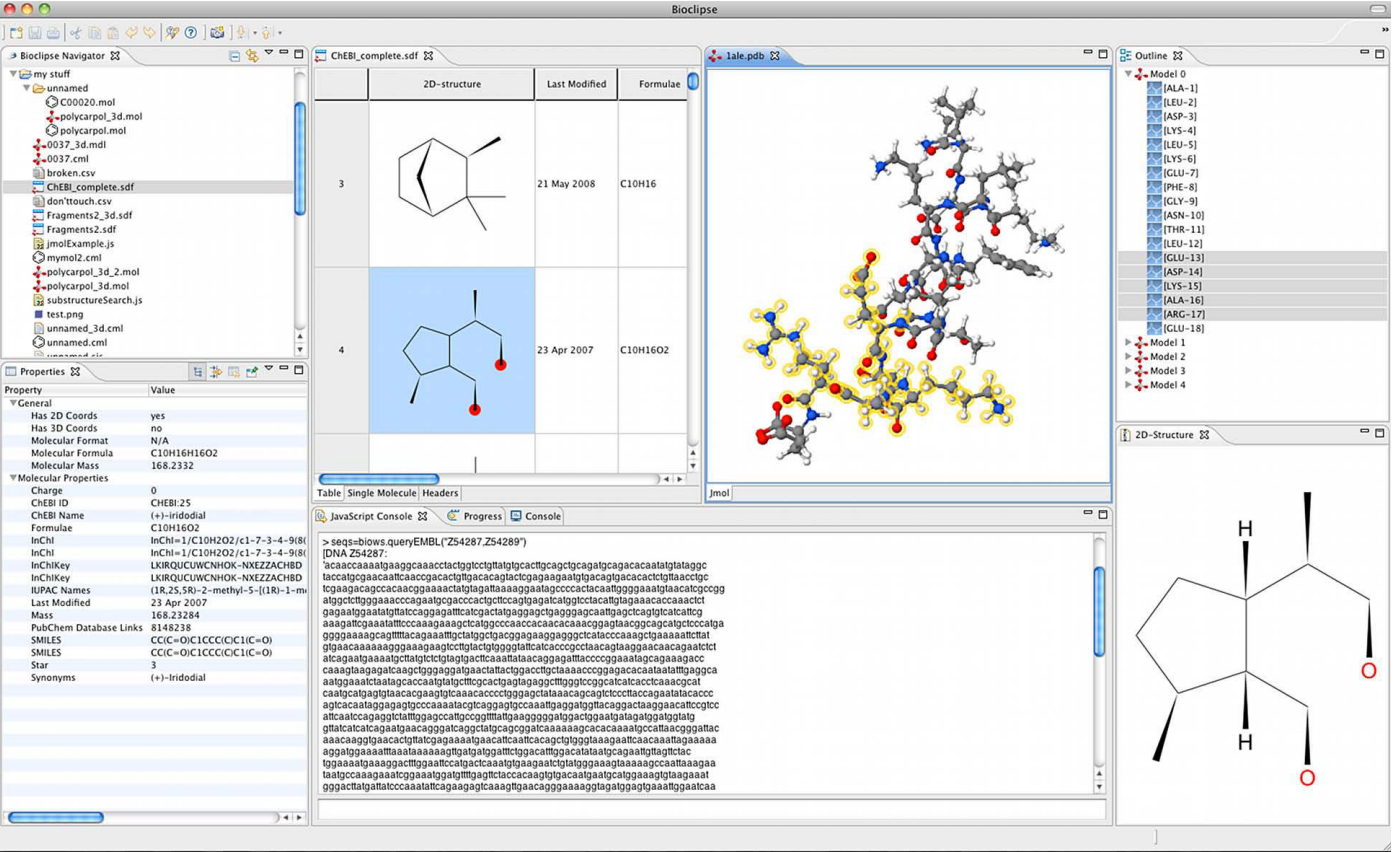
**Screenshot from Bioclipse 2**. Screenshot from Bioclipse 2 showing the new Molecules Table (top middle panel) with chemical structures and properties in a spreadsheet-like editor and the selected structure rendered in a separate 2D view (bottom right panel), and computed properties (bottom left panel). Also shown is a 3D structure where monomers are selected in the outline (top rightmost panel) and highlighted in the interactive 3D visualization component Jmol (top right panel). The Javascript console (bottom middle panel) can be used to execute scripting commands.

Cheminformatics comprises the management, analysis, and visualization of chemical structures and related information. Bioclipse 2 includes a completely rewritten version of the 2D editor JChemPaint [8] with advanced chemical editing and visualization features, a new MoleculesTable that supports editing of resources containing multiple molecules (see Figure 1), and the latest version of Jmol http://www.jmol.org for interactive 3D visualization. A domain model and extensive list of cheminformatics functionality is provided by the Chemistry Development Kit (CDK) [9] and Chemical Markup Language (CML) [10,11]; this includes for example 2D and 3D coordinates generation, SMARTS searches, distance measurements, fingerprint calculations, substructure searches, and property calculations including InChI, SMILES, and QSAR descriptors. Balloon [6] is also bundled for 3D conformer generation. The PubChem eUtils are used to query and retrieve compounds into Bioclipse, and several clients for demanding cheminformatics analyses are integrated as XMPP cloud services.

### Bioinformatics

Bioinformatics in Bioclipse 2 comprises primarily the management and analysis of sequences, proteins, and related information. In contrast to earlier versions, Bioclipse 2 features a Sequence Editor which allows for editing and visualization of sequences, including DNA, RNA, protein sequences, as well as pairwise and multiple alignments. The object model for sequence management in Bioclipse is primarily based on BioJava [12], which also provides common features like format conversions and translations. Examples of integrated Web services are Kalign for sequence alignment [13], and WSDbfetch for retrieving resources from public repositories at EBI [14].

### Spectral analysis

The spectrum feature in Bioclipse was developed with three application scenarios in mind: An input facility for spectral data in the context of a computer-assisted structure elucidation tool [15,16], the (offline) authoring of datasets for spectral databases [17], and as a tool for spectrum handling in the context of metabolomics. To these ends, a set of components were implemented which allow for parsing and editing of spectral data (NMR, MS) in open formats (JCAMP, CMLSpect), and visualization in interactive tables and graphs. Spectra can be assigned to chemical structures and this assignment can be displayed and browsed interactively by the user.

### Scripting integrative analysis

**Figure 2:**
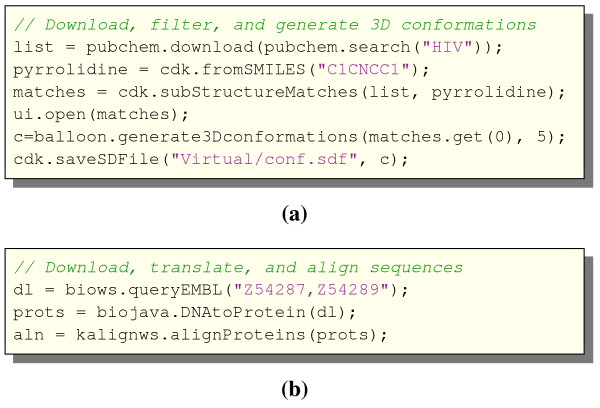
**Example scripts for Bioclipse 2**. The Bioclipse Scripting Language in Bioclipse 2 is based on Javascript with extensions contributed by plugins. Here are two example scripts that demonstrate several integrated components: **a) **PubChem is queried for compounds matching the name "HIV" using a Web service, substructures are isolated that contain pyrrolidine and opened for inspection, Balloon is used to generate 5 3D conformations of the first match, and finally the conformations are saved to an SDFile. **b) **Two nucleotide sequences are downloaded from EMBL using the WSDbfetch Web service at EBI, translated to protein using Biojava, and aligned using the Kalign Web service at EBI. The complete examples are available as gists from github with id: a) 163575 and b) 163440.

The Bioclipse plugins can contribute functionality made available via an integrated scripting environment, for example Javascript. This can be seen as a domain-specific language (DSL) for the life sciences, and we have named this Bioclipse Scripting Language (BSL). In Bioclipse 2, BSL is based on Javascript, but we envision other implementations in the future. A Javascript Console (see Figure 1) is available for executing individual commands, and a Javascript editor can be used to set up and execute scripts (see Figure 2 for two sample scripts). The mix of plugin-contributed methods with the JavaScript language has been shown to be very powerful, and makes it possible to seamlessly use integrated third party software in scripts that automate analyses.

Sharing of scripts is an important feature, as it allows for collaborators to reproduce analyses, and to provide the means to extend Bioclipse with composite functionality for a specific task. Bioclipse supports sharing of scripts via the MyExperiment [18] and Gist [19] services. Dedicated plugins in Bioclipse makes it easy to download the latest version of a script using both services. Lists of scripts are available for MyExperiment on [20] and for Gist on del.icio.us on [21].

## Conclusion

Bioclipse is an advanced integration platform for the life sciences featuring an easy-to-use workbench that delivers the latest functionality available from intuitive graphical editors and wizards, and enables users to take advantage of networked databases and online services. Experienced users will appreciate the scripting language for quickly executing powerful commands with the possibility to visualize results directly in 2D or 3D, and the easy extension of Bioclipse with new functionality. The combination of seamlessly using the GUI and the scripting language is already an appreciated feature, for example dragging and dropping resources into the scripting console to simplify scripting. To the best of our knowledge there exists no other framework, open source of proprietary, which integrates cheminformatics with bioinformatics in an extensible scripting language.

Bioclipse has an active development community, see the Bioclipse wiki http://wiki.bioclipse.net for more information. Ongoing projects include chemical and biological databases, QSAR, predictive toxicology, metabolomics, semantic data fusion, and systems biology. The Bioclipse project honors the idea of the Blue Obelisk Community [22] and promotes an open development and welcomes new developers and contributors.

## Availability and requirements

*Project name*: Bioclipse

*Project home page*: http://www.bioclipse.net

*Operating system(s)*: Platform independent

*Programming language*: Java

*License*: Eclipse Public License (EPL)

*Restrictions to use by non-academics*: None

## Authors' contributions

All authors are developers or administrators of the Bioclipse project and read and approved the final manuscript.
